# Grass-microbial inter-domain ecological networks associated with alpine grassland productivity

**DOI:** 10.3389/fmicb.2023.1109128

**Published:** 2023-01-25

**Authors:** Yingcheng Wang, Ning Dang, Kai Feng, Junbang Wang, Xin Jin, Shiting Yao, Linlin Wang, Songsong Gu, Hua Zheng, Guangxin Lu, Ye Deng

**Affiliations:** ^1^Collage of Agriculture and Animal Husbandry, Qinghai University, Xining, China; ^2^CAS Key Laboratory for Environmental Biotechnology, Research Center for Eco-Environmental Sciences, Chinese Academy of Sciences (CAS), Beijing, China; ^3^Institute of Applied Ecology, Chinese Academy of Sciences, Shenyang, China; ^4^College of Resources and Environment, University of Chinese Academy of Sciences, Beijing, China; ^5^National Ecosystem Science Data Center, Key Laboratory of Ecosystem Network Observation and Modeling, Institute of Geographic Sciences and Natural Resources Research, Chinese Academy of Sciences, Beijing, China; ^6^Institute of Marine Science and Technology, Shandong University, Qingdao, China; ^7^State Key Laboratory of Urban and Regional Ecology, Research Center for Eco-Environmental Sciences, Chinese Academy of Sciences, Beijing, China

**Keywords:** alpine grassland, bipartite network, grass-microbe associations, grass productivity, soil microbial, Three-Rivers Headwaters region

## Abstract

Associations between grasses and soil microorganisms can strongly influence plant community structures. However, the associations between grass productivity and diversity and soil microbes, as well as the patterns of co-occurrence between grass and microbes remain unclear. Here, we surveyed grass productivity and diversity, determined soil physicochemical, and sequenced soil archaea, bacteria and fungi by metabarcoding technology at 16 alpine grasslands. Using the Distance-decay relationship, Inter-Domain Ecological Network (IDEN), and Mantel tests, we investigated the relationship between grass productivity, diversity and microbial diversity, and the patterns of co-occurrence between grass and microbial inter-domain network in alpine grassland. We found the archaea richness, bacteria richness and Shannon, and fungi α-diversity were significantly negatively correlation with grass diversity, but archaea and bacteria diversity were positively correlation with grass productivity. Moreover, an increase in microbial β-diversity was observed along with increased discrepancy in grass diversity and productivity and soil variables. Variance partitioning analysis suggested that the contribution of grass productivity on microbial community was higher than that of soil variables and grass diversity, which implies that microbial community was more related to grass productivity. Inter-Domain Ecological Network showed that the grass species formed complex and stable ecological networks with some bacterial, archaeal, and fungal species, and the grass-fungal ecological networks showed the highest robustness, which indicated that soil fungi could better co-coexist with aboveground grass in alpine grasslands. Besides, the connectivity degrees of the grass-microbial network were significantly positively correlated with grass productivity, suggesting that the coexistence pattern of grasses and microbes had a positive feedback effect on the grass productivity. The results are important for establishing the regulatory mechanisms between plants and microorganisms in alpine grassland ecosystems.

## 1. Introduction

In alpine regions, due to the long-term effects of climate warming (Wu et al., 2022) and overgrazing (Wang H. et al., 2019), which further promotes the change of alpine grassland plant community (Luo et al., 2019; Mu et al., 2020). Changes in plant diversity are known to affect above- and below-ground ecosystem functioning, including diversity of below communities of other organisms (Tilman et al., 2001; Heemsbergen et al., 2004). Soil nutrient composition and microbial community activities have positive or negative effects on above-ground plants, among which the most obvious effect is the change in grassland plant productivity. Plant productivity is an important ecological property, that is closely related to the nutrient cycle, energy flow, and carbon cycle. Moreover, above-ground biomass is an important indicator of community productivity (Zhao et al., 2022). Likewise, plant species richness is thought to stabilize ecosystems, thus maintaining sustainable ecosystem functioning and services (Hautier et al., 2015; Grace et al., 2016). The evolution and development of species and ecosystems in the Qinghai-Tibet Plateau region are dynamic and changeable (Luo et al., 2002). And in consideration of its special topography and alpine climate, biomass has been used as an indicator to reflect global climate change in this region (Ni, 2000). Although alterations in soil hydrology and biogeochemical processes play roles in plant variations, microbial communities are very important biotic participators within the plant-soil feedback loop (Shang et al., 2016), and play an important ecological function in the ecosystem of the Qinghai-Tibet Plateau (Luo et al., 2002). Many previous studies focused on the variation of soil microbial community diversity with climate (Wang Z. et al., 2019), environmental heterogeneity (Wang et al., 2020), and spatial patterns in alpine grasslands on the Qinghai-Tibet Plateau (Wang et al., 2021). However, few studies have explored the association between soil microbial diversity and grass productivity in alpine grasslands in this region. There is no doubt that exploring the association between productivity and soil microbes on the Tibetan Plateau is crucial to the entire ecosystem of this region.

Previous studies on grasslands have found strong plant diversity effects on plant production (Tilman et al., 2001), but it is unclear how subterranean microorganisms affect plant production. Given the important role of soil microorganisms in determining plant production, we need to understand certain basic questions, such as their distribution patterns and the associations between microorganisms and plants, to help us predict microbial responses to changes in vegetation. Microbial distribution is controlled by environmental conditions and microbial dispersal ability (Karimi et al., 2018), by adjusting the difference of species richness or species that drive turnover and significantly impact microbial diversity (Gianuca et al., 2017). Likewise, variations that affect species richness or turnover may also lead to differences in community turnover (Soininen et al., 2018). There is a growing number of studies that the rates of community turnover associated with the sensitivity to community disturbance is also one aspect of community stability (Liang et al., 2015; Jiao et al., 2019). Soil microbial communities are involved in regulating many ecological processes. Some studies showed that negative frequency-dependent growth of plant populations detrimentally affected by soil microbial communities helps maintain plant diversity in various communities, including old fields (Pendergast et al., 2013), tropical forests (Mangan et al., 2010; Liu et al., 2021), and grasslands (Wu et al., 2022). Spatial variation in the soil microbial community can lead to variation in plant productivity (van der Heijden et al., 2008) and affect the outcome of plant restoration (Wubs et al., 2016). Thus, in-depth analysis of microbial diversity under plant variations could increase our understanding of microbial diversity and adaptability to environmental disturbances.

Associations between plants and soil microorganisms are widespread and have profound effects on plant growth and reproduction (Bever et al., 2015; Selosse et al., 2015). Despite the important role of microbial communities for grassland plants, there has a little examination of the dynamic interrelationships between plant and community composition. Soil microbes and plants not only provide each other with beneficial substances, and form a reciprocal symbiose, but also compete for environmental resources (Kuzyakov and Xu, 2013; Averill et al., 2014; Alejandra et al., 2021). These associations and feedbacks form an important link between the below- and above-ground ecosystem components. Soil microbes can alter plant community dynamics (Kandlikar et al., 2019), while the differential response of plant species to soil microbes can contribute to negative-frequency dependent plant population dynamics that may promote diversity (Mangan et al., 2010; Bever et al., 2015). Hassani et al. (2018) found that the interactions between subterranean microorganisms and above-ground plants contributed to community structure and plant health. Feng et al. (2019) proposed a workflow to construct interdomain ecological networks (IDENs) between multiple plants and various microbes, and utilized plant ecological survey data and microbial sequencing datasets from 30 widely distributed latitudinal forest sites, covering a distance of over 2,500 km in China, to generate regional IDENs. It was found that plant species exhibited a strong preference for specific microbial groups, and the plant-forest link distributions showed the geographical distribution of plants had higher endemicity than that of microorganisms. Therefore, we wanted to know what is the relationship between grass plants and microbial communities in the alpine grasslands of the Qinghai-Tibet Plateau, and whether the impact of this association on plants is reflected in plant productivity.

As an extremely remote area of the Qinghai-Tibet Plateau, the Three-Rivers Headwaters (TRH) region of Qinghai Province is one of the most fragile ecosystems in China. It is the key to ensuring the water resource security of the Lancang River, Yellow River and Yangtze River basins and to the sustainable development of the ecosystem (Wang and Wang, 2019). Environmental factors such as soil pH and precipitation were found to have significant correlations to differences in soil bacterial diversity and community composition on the Tibetan Plateau (Chu et al., 2016; Duckworth et al., 2019), while soil fungal diversity was significantly related to plant diversity (Yang et al., 2017). Therefore, we chose an areas 2,121 km across, encompassing alpine swamp meadow, alpine meadow, alpine steppe and temperate steppe ecosystems, and analyzed the community diversity and composition distribution pattern soil archaea, bacteria, and fungi, as well as the relationship between grasses and microbes. Specifically, we addressed the following questions: (i) What is the relationship between microbial diversity and grass diversity? (ii) What is the relationship between microbial diversity and grass productivity? (iii) What are the ecological networks between grasses and microorganisms in TRH?

## 2. Materials and methods

### 2.1. Study area

The study area spans a 2,121 km transect that encompasses arid to mesic grasslands in the TRH region (95.565°E~100.919°E, 33.202°N~35.929°N), including alpine swamp meadow (ASM), alpine meadow (AM), alpine steppe (AS), and temperate steppe (TS) with decreasing altitude (Altitude: 4,446~3,233 m) and increasing mean annual temperature (MAT: −0.47~3.66°C) (Supplementary Figure S1, Table 1). The alpine swamp meadow was the richest in plant species, dominated by perennial moisture-loving herbs including *Kobresia littledalei, Kobresia capillifolia, Carex moorcroftii*, etc. The alpine meadow had the most abundant plants in the TRH region with plants that require a moderate amount of moisture, dominated by *Kobresia pygmaea, Poa pratensis, Potentilla nivea*, etc. The alpine steppe was composed of perennial herbaceous plants, which have drought- and cold-resistance characteristics, including *Stipa purpurea, Oxytropis deflexa, Leontopodium nanum*, etc. The temperate grassland had the highest temperature, and was composed of perennial drought-tolerant plants, dominated by *Stipa purpurea Griseb, Artemisia sieversiana, Orinus kokonorica*, etc.

**Table 1:** Changes of environmental factors in different grassland types.

**Environmental factors**	**Variables**	**Alpine swamp meadow (ASM)**	**Alpine meadow (AM)**	**Alpine steppe (AS)**	**Temperate steppe (TS)**
Soil physical–chemical factors	pH	7.21 ± 0.57^b^	7.02 ± 0.58^b^	8.07 ± 0.11^a^	8.12 ± 0.17^a^
	CEC [cmol(+)/kg]	25.51 ± 14.59^a^	27.09 ± 15.94^a^	6.60 ± 3.99^b^	11.94 ± 2.32^b^
	OM (g/kg)	86.05 ± 22.55^b^	131.30 ± 75.35^a^	44.43 ± 25.66^c^	46.25 ± 15.50^c^
	TN (g/kg)	3.40 ± 1.26^ab^	4.35 ± 1.58^a^	1.84 ± 1.06^c^	2.55 ± 4.97^bc^
	TP (g/kg)	0.57 ± 0.22^b^	0.73 ± 0.12^a^	0.43 ± 0.09^c^	0.71 ± 0.04^a^
	TC (%)	5.3 ± 1.55^b^	8.55 ± 4.62^a^	3.55 ± 1.59^b^	3.31 ± 0.85^b^
	SMC (%)	50.75 ± 18.72^a^	30.31 ± 11.39^b^	14.45 ± 7.52^c^	15.26 ± 11.83^c^
Grass biophysical variables	H (cm)	11.49 ± 4.16^b^	4.18 ± 2.73^c^	8.14 ± 8.40^bc^	20.49 ± 5.09^a^
	CD (%)	93.00 ± 11.91^a^	76.75 ± 11.74^b^	55.44 ± 15.56^c^	49.25 ± 20.76^c^
	S	9.67 ± 2.25^a^	11.92 ± 5.08^a^	6.56 ± 1.54^b^	5.42 ± 2.67^b^
	FB (g/m^2^)	388.13 ± 223.69^a^	361.10 ± 263.06^a^	216.49 ± 227.82^a^	266.03 ± 151.47^a^
	DB (g/m^2^)	187.20 ± 111.52^a^	124.70 ± 76.41^ab^	107.80 ± 85.87^b^	136.73 ± 58.73^ab^
Temperature	MAT (°C)	−0.47 ± 0.93^b^	−0.19 ± 0.75^b^	0.34 ± 1.69^b^	3.66 ± 0.20^a^
Geography	Altitude (m)	4,445.67 ± 234.42^a^	4,187.58 ± 122.47^b^	4,014.89 ± 390.24^b^	3,232.25 ± 67.45^c^

The Soil pH (pH), cation exchange capacity (CEC), organic matter (OM), total nitrogen (TN), total carbon (TC), total phosphorus (TP), soil moisture content (SMC), the grass height (H), coverage degree (CD), species(S), fresh biomass (FB), dry biomass (DB), and the mean annual temperature (MAT). Lowercase letters indicate significant differences between grassland types at the 0.05 level.

### 2.2. Soil and grass sampling

A total of 48 soil and grass samples were collected respectively from 16 sites in the TRH region, including two from ASM, four from AM, six from AS and four from TS in August 2018. Grass samples were randomly sampled using the quadrate of 0.5 × 0.5 m^2^, and each site had three replicates with a minimum distance of 200 m. The natural height of all grasses in the quadrat was measured 10 times, and the average height (H) of all grasses was calculated. The grass coverage degree (CD) was measured by the pinprick method, and the number of species in the quadrate was tallied. All the above-ground of the grasses in the quadrat were cut off with scissors, respectively put into envelope bags and marked (Fang et al., 2009). Soil samples were also collected in triplicate at each site. Soil samples were randomly taken from the topsoil (0–15 cm) within a 5 m radius of the grass sampling square. The three subplot replicates were thoroughly mixed to create a composite sample for each site (Fang et al., 2009; Li et al., 2021). Then all soil samples were divided into two portions. One portion for DNA analysis was transported in an ice box to the laboratory and stored at −80°C immediately upon arrival, while the other portion was air-dried for physicochemical analyses. All soil samples were sieved through a 2 mm mesh with visible roots removed before laboratory analysis.

### 2.3. Analysis of soil physical and chemical properties, altitude, and MAT data

Global Positioning System (GPS) was used to identify the geographic location of each site. The mean annual temperature (MAT) of each site was collected from 2005 to 2015 records using the geographic ordinates (www.worldclim.org). Standard methods for assessing soil physicochemical properties was used to acquire a suite of environmental parameters including pH (Dai et al., 2018), cation exchange capacity (CEC) (Hendershot and Duquette, 1986), organic matter (OM) (Walkley and Black, 1934), total nitrogen (TN), total carbon (TC) (Vario EL III; Elementar, Hanau, Germany), total phosphorus (TP) (Cai et al., 2017), and soil moisture content (SMC) (Reynolds, 1970). All measurements were performed at were determined to reflect the corresponding environmental conditions by the Institute of Soil Science, Chinese Academy of Sciences.

### 2.4. DNA extractions and high-throughput amplicon sequencing

Total DNA from soil samples was extracted using the MoBio PowerSoil DNA isolation kit (MoBio Laboratories, Carlsbad, CA, USA) according to the manufacturer's protocol. For soil prokaryotic communities, we used the universal primer set 515F/806R (515F, 5′-GTGCCAGCMGCCGCGGTAA-3′ and 806R, 5′-GGACTACHVGGGTWTCTAAT-3′) for the V4 region of the 16S rRNA gene (Li et al., 2022). For soil fungal communities, we used the universal primer set gITS-7F/4R (7F, 5′-GTGARTCATCGARTCTTTG-3′ and 4R, 5′-TCCTCCGCTTATTGATATGC-3′) targeting the fungal ITS2 (Internal Transcribed Spacer) gene (Lu et al., 2013). 10 bp barcode sequences were added to the ends of the forward and reverse primers to distinguish all soil samples.

The PCR amplification was carried out on SelectCycler™II Thermal Cycler (Select BioProducts), with 48 DNA samples for 16S rRNA gene and ITS, in a 50 μL reaction system, including 5 μL of 10 × PCR buffer, 4 μL of dNTPs, 0.5 μL of Taq DNA polymerase (TaKaRa Biotech, Beijing, China), 1.5 μL of 10 mM each primer, 20~30 ng of genomic DNA, and ddH_2_O up to 50 μL. The PCR program consisted of denaturing for 5 min at 96°C, followed by 30 cycles and 33 cycles of 20 s at 96°C, 25 s at 57°C, 45 s at 68°C for 16S rRNA gene and ITS respectively, and final extension at 68°C for 10 min. The PCR products were visualized on a 1% agarose gel and the target bands were extracted using E.Z.N.A.™ Gel Extraction Kit (Omega Bio-tek, Norcross, GA, USA). The PCR products were quantified using a Qubit fluorometer (Life technologies Holdings Pte Ltd, Singapore) and pooled in an equal ratio (100 ng each). The mixture was used for library construction and then sequenced on the Illumina Hiseq platform using a PE250 kit (2 ^*^ 250 bp) at Magigene Biotechnology Co., Ltd. (Guangzhou, China).

### 2.5. Processing of sequence data and Statistical analysis

Raw DNA sequences were processed on a Galaxy pipeline (http://mem.rcees.ac.cn:8080). The raw reads were assigned to different samples according to their barcodes, allowing for one mismatch. FLASH program (Magoč and Salzberg, 2011) was used to combine the forward and reverse sequences. Quality control criteria consisting of average quality score >20, minimum length of 140 bp for 16S, 300 bp for ITS. As a result, we obtained a total of 4,324,237 and 1,355,773 high-quality sequences for prokaryotes and fungi, and the corresponding OTUs were clustered using the UPARSE pipeline (Edgar, 2013) at 97% sequence similarity per sample. Here, the total number of samples obtained is 48, but, in the laboratory experiment, the number of reads obtained from sequencing of 7 sample in ITS was <10,000. After the second experiment, it was still unqualified, so those samples were excluded from subsequent analysis and statistical calculations. The OTUs identified as archaea and bacteria in the 16S OTU table, fungi OTU in the ITS OTU table. To correct for sampling error, reads counts from 48 samples were normalized to 20,828 for 16S gene, 41 samples were normalized to 10,140 for ITS gene.

The differences in environmental factors among the four grassland types were determined using a non-parametric test. The Margalef, Shannon, and Pielou indices were used to measure the α-diversity of the grass community in different grassland types. In this study, the soil microbial α-diversity was estimated using Shannon Index, Observed Richness and Pielou Index. Spearman's method was used to calculate the correlation between microbial alpha diversity and grass diversity, productivity, and soil variables. Non-metric Multidimensional scaling (NMDS) based on Bray-Curtis distance was used to compare community structure in different grassland types. While the difference of microbial communities among grassland types were calculated by Permutational Multivariate Analysis of Variance (PERMANOVA) based on Bray-Curtis. Canonical correlation analysis (CCA) was mainly used to calculate the correlation between microbial communities of different grassland types and environmental factors. To predict microbial function, Functional Annotation of Prokaryotic Taxa (FAPROTAX v.1.2.4) were used to create a archaea and bacteria function heatmap (Louca et al., 2016), FUNGuild were used to create a fungi function heatmap (Nguyen et al., 2016), functional abundance >0.1% was selected for visualization. In addition, microbial distance-decay relationships were estimated from microbial dissimilarity (Bray-Curtis dissimilarity) and geographic distance or divergence of grass productivity (i.e., grass Height, CD, S, FB, and DB) and diversity (i.e., Margalef, Shannon, and Pielou) (Euclidean distance based on measured plant productivity and diversity) using the ‘vegan' R package (Dixon, 2003). The significance of slope of the two curves was tested using the “lsmeans” R package (Lenth, 2016), using the analysis of variance (ANOVA) to test. The variance partitioning analysis (VPA) was used to determine the proportion of changes in microbial (i.e., archaea, bacterial, and fungi) community structure explained by grass productivity (i.e., CD, DB, and S), diversity (i.e., Margalef, Shannon, and Pielou), and soil variables (i.e., soil pH, TN, TP, and SMC).

To elucidate the associations between grasses and soil microorganisms, bipartite ecological networks were constructed using the iNAP workflow (http://mem.rcees.ac.cn:8081) with a SparCC approach specifically designed to process the constituent data (Feng et al., 2017, 2022; Zhang et al., 2022). Uncorrelated associations in the matrix were filtered at a threshold of 0.3 and significance of 0.05 to generate a bipartite network. We then utilized the new mathematical framework inference of direct and indirect relationships (iDIRECT) to quantitatively infer direct dependencies in the association networks, which allowed the inference of direct and indirect relationships based on the transitivity of effective copules (Xiao et al., 2022). Next, we used the latest environmental filtering or diffusion restriction (LTED) link test to eliminate connections in the network related to environmental factors and geographical distance (Yuan et al., 2021), and finally we obtained an association networks that best represented the variation between the species themselves. The topological properties were calculated by IDENs to show whether grassland grass and soil microbial association. To understand whether and how grassland types affected the stability of the constructed bipartite networks, robustness and vulnerability was used to characterize the stability of the networks and their embedded members (Deng et al., 2012; Yuan et al., 2021). Finally, Partial mantel tests were performed between grass-microbial bipartite network connectivity and grass productivity, diversity and soil variables (Deng et al., 2012). The obtained network, where nodes and edges represent the saliency interactions between two species, plant and microbial, respectively, was visualized in Gephi (0.9.2) (Bastian et al., 2009) and Cytoscape (3.8.2) (Shannon et al., 2003).

## 3. Results

### 3.1. Relationship between grass productivity and diversity and soil variables

Through investigation and detection, we found that grass biophysical and soil variables changed significantly among different grassland types (Table 1). For grass biophysical variables, TS had the highest average grass height (H), but lower grass cover degree (CD) than ASM and AM, which was mainly due to the predominance of Gramineae plants. ASM had the highest grass CD, fresh biomass (FB), and dry biomass (DB). The soil of CEC, TN, TP, TC, and OM were the highest in AM, followed by ASM, and lowest the AS. Furthermore, from ASM to TS, soil pH increased and soil moisture decreased, these results indicated that the soil nutrient composition of meadow was better and more fertile than steppe. We found with the decrease in altitude, MAT increased significantly (Table 1). On the other hand, the correlation between grass productivity, diversity, and soil variables showed that grass CD and FB were significantly negatively correlations with soil pH, grass CD was significantly positively correlation with soil CEC, OM, TN and SMC (Supplementary Table S1), grass S was significantly positively correlation with soil TP and TC. The grass diversity was significantly positively correlation with soil pH, Margalef was negatively correlated with soil CEC, OM, and SMC, Shannon was significantly negatively correlation with soil CEC, OM, TN, and SMC, Pielou was significantly negatively correlation with soil CEC, TN, TP, and SMC (Supplementary Table S1).

### 3.2. Relationship between grass productivity and diversity and microbial α-diversity

**Figure 1:**
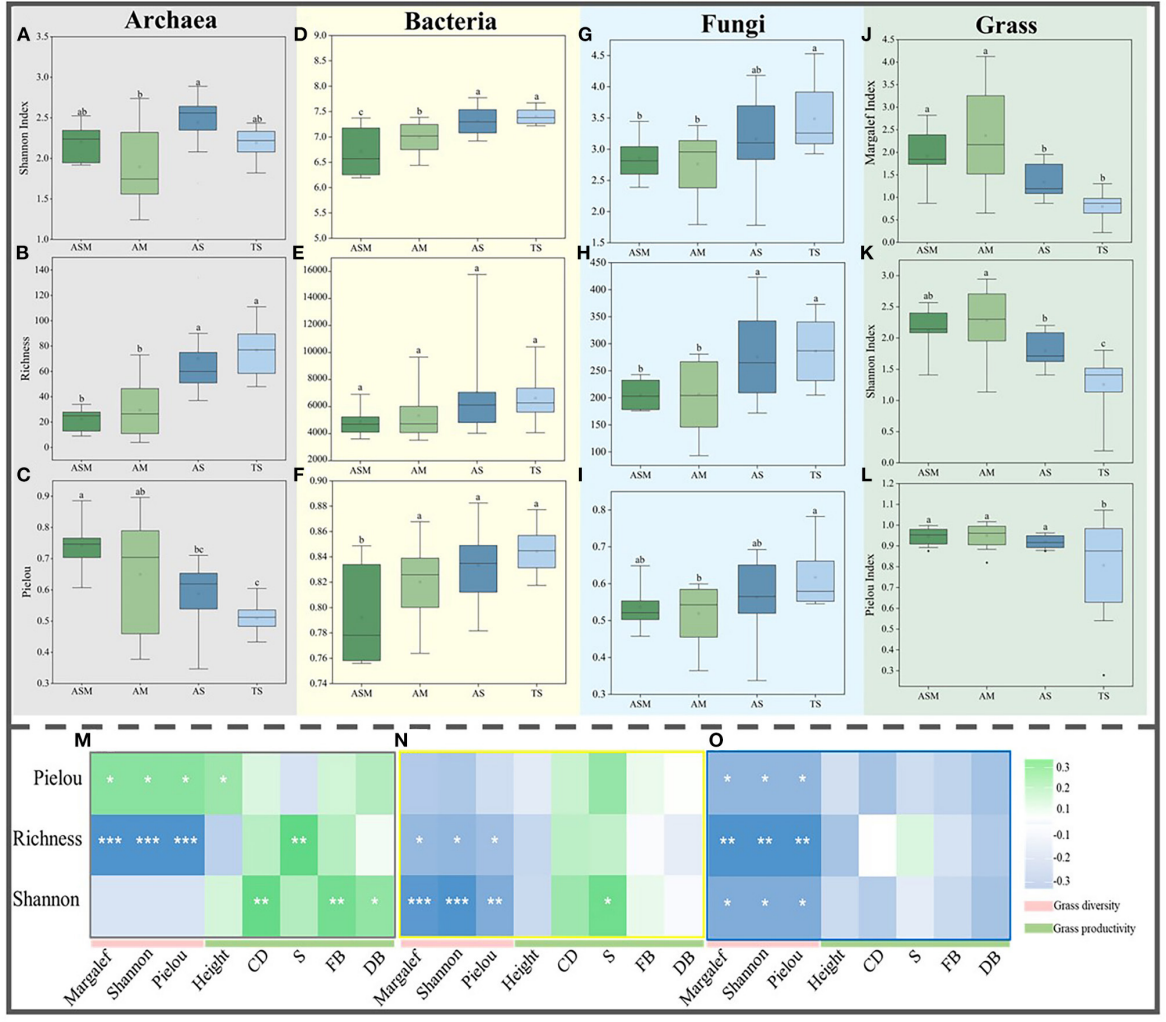
The α-diversity of microbial and grass communities. The Shannon index **(A)**, Richness **(B)**, and Pielou index **(C)** of archaea. The Shannon index **(D)**, Richness **(E)**, and Pielou index **(F)** of bacteria. The Shannon index **(G)**, Richness **(H)**, and Pielou index **(I)** of fungi. The Margalef **(J)**, Shannon index **(K)**, and Pielou index **(L)** of grasses. The correlation between grass diversity and productivity and archaeal **(M)**, bacterial **(N)**, and fungal **(O)** α-diversity. Different lowercase letters indicate significant differences between grassland types at the 0.05 level. The correlation between microbial alpha diversity and grass diversity and productivity at 0.001***, 0.01**, and 0.05* level.

By calculating the α diversity of grass and microbial communities, the results showed that there were significant differences between the grass and microbial communities of the four grassland types in the TRH region. For grass α-diversity, the grass Margalef, Shannon, and Pielou indexes showed the same trend, with highest in AM, followed by ASM and lowest in TS (Figures 1J–L). For microbial α-diversity, archaeal richness showed an increasing trend (Figure 1B), but the Pielou index displayed a decreasing trend (Figure 1C). Bacterial and fungal Shannon, richness, and Pielou indices showed increasing trends (Figures 1D–I). The correlation between grass α-diversity, productivity, and microbial α-diversity showed that the α-diversities of the archaeal richness, bacterial richness and Shannon, and fungal communities were all significantly negatively correlated with grass α-diversity (Figures 1M–O), but the archaeal Pielou was significantly positively correlation with grass α-diversity (Figure 1M). The archaeal richness and Shannon and bacterial Shannon were significantly positively correlation with grass productivity (Figure 1M–O), but fungal community α-diversity was negatively correlation with grass productivity (Figure 1O). Meanwhile, the archaea Shannon, richness, and bacteria Shannon were significantly positively with soil physicochemical variables, and significantly negatively with soil pH (Supplementary Table S1).

We investigated the relative abundance of microbial phyla and all grass species in the different grassland types (Supplementary Figure S2), and found the dominant microbial phyla varied significantly among the four grassland types (Supplementary Table S2). Additionally, different grassland types also possessed different microbial functions as indicated by FAPROTAX and FUNGuild analyses (Supplementary Figure S3). Based on the Bray-Curtis matrix, the results revealed that soil archaea, bacteria, fungi, and grass communities were significantly different among the grassland types (PERMANOVA, *P* < 0.001, Supplementary Figures S4A–D, Table S3). The environmental factors that had the strongest impact on the archaea and bacteria communities were soil TP, S and DB (Supplementary Figures S4E, F), only CD had the strongest impact on fungi community (Supplementary Figure S4G), while the factors that had the strongest impact on the grass community were soil TN, SMC, soil pH, and grass S and CD (Supplementary Figure S4H). These results indicated soil microbial communities differed significantly among the grassland types and that grasses had an impact on the microbial community.

### 3.3. Relationship between grass productivity and α-diversity and microbial β-diversity

**Figure 2:**
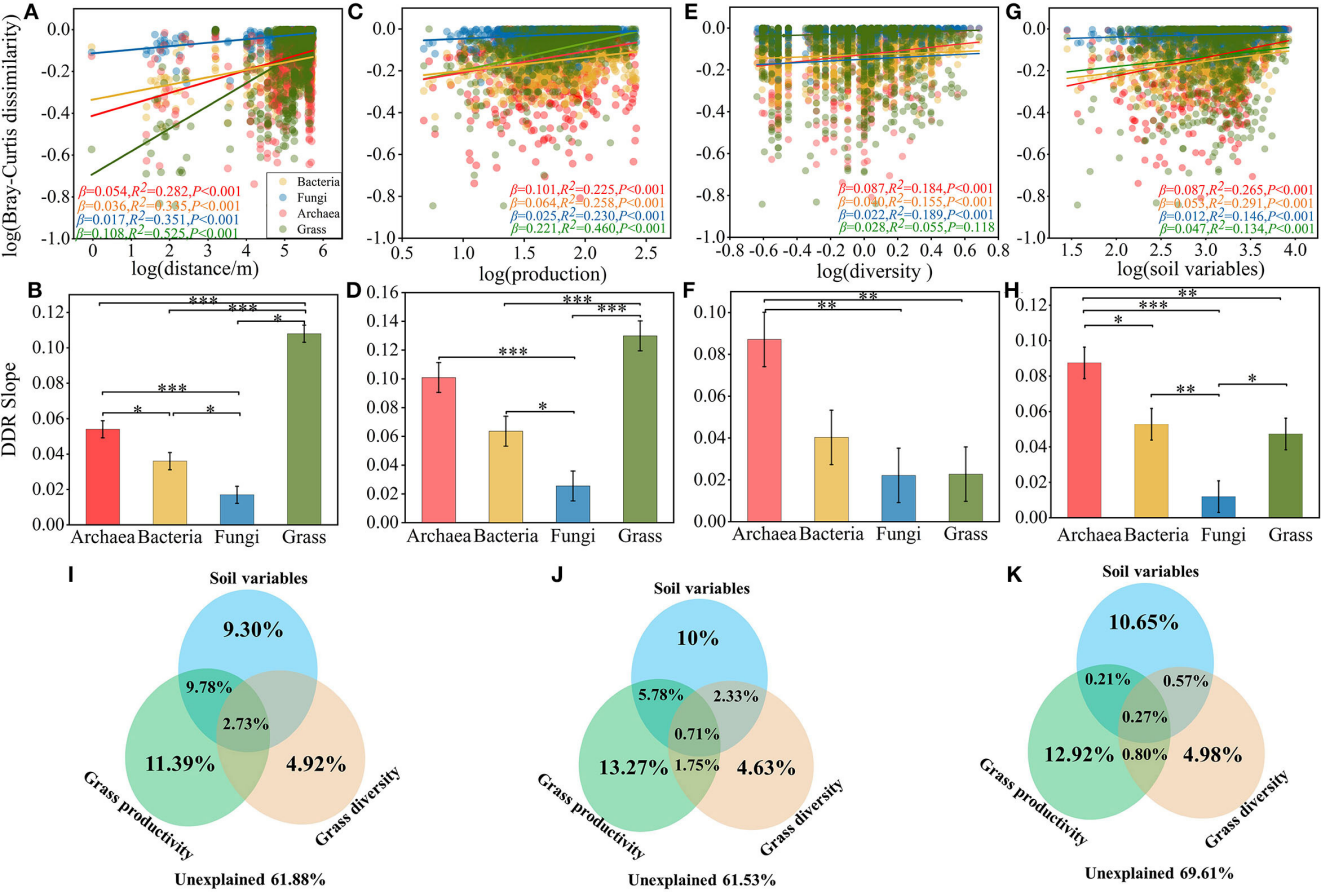
Distance decay relationships among geographical distance, grass productivity, diversity and microbial communities. **(A)** DDRs based on Bray-Curtis dissimilarity along geographic distance for each organism group. **(B)** Slope test of microbial community and geographical distance. **(C)** Dissimilarity between microbial communities and grass production. **(D)** Slope test of microbial community and grass production. **(E)** Dissimilarity between microbial communities and grass diversity. **(F)** Slope test of microbial community and grass diversity. **(G)** Dissimilarity between microbial communities and soil variables. **(H)** Slope test of microbial community and soil variables. Variation partitioning analysis based on CCA illustrating the effects of grass productivity/diversity and soil variables factors on the archaea **(I)**, bacteria **(J)**, fungi **(K)** communities. Slope of ANOVA test: *P < 0.05, **P < 0.01, and ***P < 0.001.

To further verify the effect of microbial community beta diversity on grass productivity and diversity, we evaluated the spatial distribution of archaea, bacteria, fungi, and grass, and the associations between grass diversity, productivity, and microbial β diversity (Figure 2). The community dissimilarity vs. geographic distance for each pairwise set of samples displayed a significant distance decay relationship for each organism group (Figure 2A). Meanwhile, the slopes of distance decay that were estimated by linear regression models varied across the different organism groups (Figure 2B). The slope of grasses (β = 0.108, *P* < 0.001) was significantly steeper than archaea (β = 0.054, *P* < 0.001), bacteria (β = 0.036, *P* < 0.001), and fungi (β = 0.017, *P* < 0.001). The slope of the distance decay curve of grasses was significantly higher than that of the microbial communities (Supplementary Figure S5). The trends for ASM and AM (Supplementary Figures S5A, B), and of AS and TS, were similar (Supplementary Figures S5C, D), but the change trend of spatial conversion of bacteria and archaea in meadow and steppe were the opposite. This result suggests that changes in archaea and bacteria are more susceptible to habitat influences. In addition, we found a significant correlation between microbial β diversity and differences in grass productivity and diversity (Figures 2C–F). As the discrepancy in grass productivity and diversity, the species turnover rates increased and communities became more dissimilar (Figures 2C, E), and the slope of relationship was lower in fungi, followed by bacteria and archaea (Figures 2D, F), and increasing discrepancy in soil variables also make communities became more dissimilar (Figures 2G, H). These results indicated that grass diversity and productivity and soil variables are positively correlated with microbial β-diversity, but which variable was more correlated with microbial community?

In order to further clarify the contribution of grass α-diversity, productivity, and soil variables to microbial communities, the results of VPA showed that the community variation of archaea (38.12%), bacteria (38.47%) and fungi (30.39%) were explained by grass productivity, diversity and soil variables (Figures 2I–K). Specifically, in the archaea, bacteria, and fungi communities of alpine grassland soil, soil variables explained 9.30, 10, and 10.65%, grass productivity explained 11.39, 13.27, and 12.92%, and grass diversity explained 4.92, 4.63, and 4.98% of the community variations, respectively. These results indicated that the contribution of grass productivity on microbial community was stronger than that of soil variables and grass diversity, which implies that grass productivity was more correlation with microbial community.

### 3.4. Grass-microbial ecological network correlated with grass productivity

**Figure 3:**
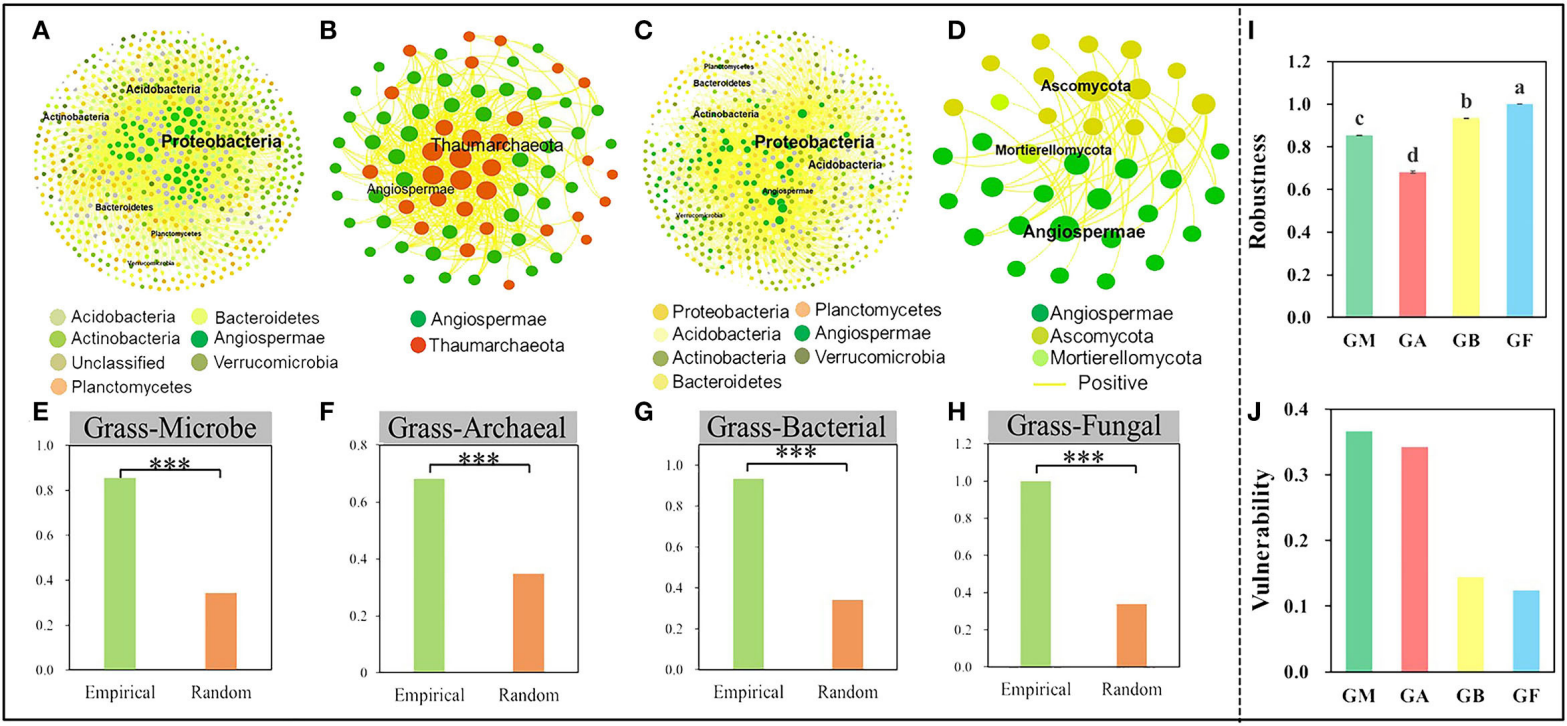
Network interactions between microorganisms and grasses. The interactions of all microorganisms and grasses **(A)**, archaea and grasses **(B)**, and bacteria and grasses **(C)**. **(D)** The interactions of fungi and grasses. Robustness of all microorganisms-grass **(E)**, archaeal-grass **(F)**, bacterial-grass **(G)**, and fungal-grass networks **(H)**. The robustness **(I)**, vulnerability **(J)** of grass and microbial, archaeal, bacterial, and fungal networks. The robustness of empirical and random networks was significantly at the 0.001*** level.

To discern the IDEN pattern in the natural grassland ecosystems, we attempted to disentangle the effects of grassland succession on the ecological interactions among different organism groups. Therefore, bipartite networks between microbes and grasses and between the two microbial groups were constructed (Figure 3, Supplementary Figure S6), and their network topological and structural indices were measured. The bipartite networks showed some basic network topological features (Supplementary Table S4). Compared to the microbe-microbe networks, the connectivity, average cluster coefficient, and nestedness of grass-microbe networks were increased by 52, 53.4, and 41.5% respectively (Supplementary Table S4). Furthermore, all links in the grass-microbe bipartite networks were positive, indicating that there was a reciprocal relationship between grasses and microorganisms in alpine grasslands, and those microorganisms co-existing with grasses could promote grass growth (Figures 3A–D). In addition, we calculated the robustness of the networks, was significantly higher than that of the random networks, indicating that the bipartite networks of grasses and microorganisms was stable (Figures 3E–H). Moreover, we found that the robustness of grass-fungal network was the highest (Figure 3I), and the vulnerability was the lowest (Figure 3J), this indicated that soil fungi could better co-coexist with aboveground grass in alpine grasslands. In order to further evaluate the relationship between grass productivity, diversity and the grass-microbial ecological network, we used partial Mantel to test the correlation between grass productivity, soil variables and network connectivity (Table 2). The results showed that there was a significant correlation between grass productivity, diversity and network connectivity, but not the soil variables.

**Table 2:** The partial mantel test of bipartite networks connectivity with grass productivity, diversity, and soil variables.

**Partial mantel**		**Grass-microbe**	

		***r***	***p***
Grass productivity	**Height**	−0.089	0.999
	**CD**	0.238	**0.001**
	S	−0.050	0.982
	**FB**	0.303	**0.001**
	**DB**	0.385	**0.001**
Grass diversity	**Margalef**	0.187	**0.001**
	**Shannon**	0.141	**0.004**
	Pielou	0.011	0.266
Soil variables	PH	−0.022	0.832
	CEC	−0.134	1.000
	OM	−0.194	1.000
	TN	−0.164	1.000
	TP	−0.024	0.766
	TC	−0.141	1.000
	SMC	0.408	0.001

## 4. Discussion

The TRH region is an important ecological resource for China, and its ecological environment has an important impact on environmental changes not just in China, but for the entire Asian continent (Liu et al., 2008). Elucidating the composition, distribution, and associations between grasses and microorganisms, including archaea, bacteria and fungi, could be important to understanding the relationship of grasses and microorganisms in alpine grasslands, and further provide a theoretical basis for the stability of alpine grassland ecosystems. Previous studies have mainly focused on the microbial community of the TRH region (Zhang et al., 2006; Xiong et al., 2012; Shi et al., 2016; Zhang K. et al., 2016; Li et al., 2017), while generally neglecting their associations with the aboveground vegetation. The current study investigated the relationship between vegetation (i.e., grass diversity and productivity), soil variables and belowground microorganisms in the TRH. It further revealed the belowground factors associated with the change in grass productivity in this alpine ecosystem.

Due to intense human activities, the ecological environment of alpine grassland ecosystems have been greatly disturbed (Jing et al., 2022). Moreover, climate and land use types have changed, leading to shifts in vegetation types (Yannikos et al., 2014; Raiesi and Beheshti, 2015), creating further significant impacts on the composition, structure and functions of the soil microbial communities (Han et al., 2007; Jangid et al., 2011). The results of the current study revealed that, with grass variations from the alpine swamp meadow to the steppe, soil nutrients, plant biomass and coverage all decreased, in accordance with the increasing temperature and decreasing altitude (Table 1). This result was consistent with previous studies, which showed that there were significant differences in vegetation community structure among different grassland types, including species, dominant species, and vegetation height, followed by differences in soil environment (Zhang Y. et al., 2016; Wu et al., 2022). Furthermore, our results showed that soil microbial and grass diversity displayed opposing trends in different grassland types (Figure 1), indicating that the belowground microbial diversity did not coincide with aboveground grass diversity in the TRH region. This result is contrary to previous findings that microbial α diversity had a strong consistency with grass diversity (Jing et al., 2015; Chen et al., 2021). The productivity of grass was positively correlated with archaea and bacterial diversity, but negatively correlated with fungal community, which may indicate that different biological groups have different effects on the growth of grass (Figures 1M–O). At a certain spatial scale, high microbial diversity is not necessarily consistent with grass diversity and productivity, which might be driven by different environmental filtering procedures for grasses and microbes (Jing et al., 2022). This is especially true in the Qinghai-Tibet Plateau, where the environment is heterogeneous and climate changes are complex (Wu et al., 2014; Sun et al., 2016).

Community similarity with increasing geographic distance is a well-described pattern of biodiversity, from macro-organisms to microorganisms (Soininen et al., 2007; Morlon et al., 2008). In this study, we observed a steeper turnover slope in the grass community (β = 0.108, *P* < 0.001) than bacterial (β = 0.036, *P* < 0.001), archaeal (β = 0.054, *P* < 0.001) and fungal communities (β = 0.017, *P* < 0.001), indicating aboveground species are likely more limited by dispersal and environmental filtering. Then, we further assessed the relationship between community dissimilarity and grass diversity, productivity, and soil variables in the TRH, and the results showed that dissimilarity of the microbial communities increased with the increasing discrepancy in grass diversity, productivity, and soil variables (Figures 2C–H). Here, we found that the turnover rate of archaea species is higher. We guess that the environment of alpine grassland has changed due to the influence of global warming in recent years, which leads to the change of the archaea community. This is a question worth of further study, especially in the alpine region of Qinghai-Tibet Plateau. Furthermore, we measured the contribution between grass productivity, diversity, and soil variables and microorganisms by variance partitioning analysis, the results showed that the contribution of grass productivity to microbial communities was greater than that of soil variables and grass diversity (Figures 2I–K). Here, the discrepancy in plant diversity, productivity, and soil variables indicated habitat heterogeneity. Environmental heterogeneity, as represented by a large discrepancy in vegetation diversity, productivity, and soil variables, creates numerous new ecological niches, and this is what accelerates the spatial transformation of species. On the other hand, soil microbial turnover promotes the increase of plant diversity and productivity through plant-soil feedback regulation (Bartelt-Ryser et al., 2005). In a high-diversity environment, some specific species, due to spatial turnover, increase complementarity among species (e.g., resource use) *via* spatial redistribution and thereby increase plant productivity improvement in species-rich communities (Thakur et al., 2021). Previous studies have suggested that an increase in niche differentiation (e.g., *via* resource partitioning) in diverse plant communities drives an increase in mixture performance over time (Cardinale et al., 2007; Hector and Wilby, 2009). But what is driving this observed increase in niche differentiation among grass?

Recently, researchers revealed that the belowground microbial consortia and their interactions with aboveground plants can indirectly switch ecosystem functions and plant productivity through resource utilization in different ecological niches (Thakur et al., 2021). Associations between grasses and soil microbial can strongly influence plant diversity and community dynamics. Until now, our understanding of the association between microbes and grasses had been largely limited to linear descriptive analysis, such that the relative importance of cooperation or competition between microbes and grasses has remained largely unresolved. Microorganisms develop interactions within certain ecological niches, such as commensalism, competition, and predation (Barberan et al., 2012; Faust and Raes, 2012), which can be revealed by microbial IDEN analysis (Feng et al., 2019, 2022; Zhou et al., 2021; Zhang et al., 2022). Synergistic interactions between microbes and plants are helpful to promote the sustainable development of grassland plant productivity and stability of grassland ecosystems. In this study, we used the IDEN method to interpret the interaction relationship between microorganisms and grasses, and the results showed that compared to the microbe-microbe networks, connectivity was 52% higher under the grass-microbial networks (Supplementary Table S4), and IDENs of the current study had slightly higher connectivity than previously studied plant-fungal (0.500 vs. 0.072) (Toju et al., 2014) and plant-bacterial (0.500 vs. 0.078) (Feng et al., 2019) networks, indicating a higher proportion of observed connections between grasses and microorganisms. In addition, the average cluster coefficient and nestedness in the grass-microbe networks were significantly higher than microbe-microbe networks (Supplementary Table S4), indicating that generalist grasses are associated with specialist microorganisms. Surprisingly, all of the links in grass-microbial bipartite networks were positive (Figures 3A–D). In addition, the robustness of the bipartite networks of grasses and microorganisms show that empirical network was higher than the random network, indicating that it was extremely stable (Figures 3E–H). A previous study indicated that network stability was strongly correlated with network complexity (Yuan et al., 2021), and environmental degradation could decrease microbial community stability within disturbed disturbance ecosystems (Wu et al., 2021). Moreover, we found that the robustness of grass-fungal network was the highest (Figure 3I), and the vulnerability was the lowest (Figure 3J), this indicated that soil fungi could better co-coexist with aboveground grass in alpine grasslands. In the fungal community, especially some arbuscular mycorrhizal fungi form a widespread symbiosis with plants that shapes the composition of plant communities and the functioning of ecosystems (Davison et al., 2021).

In this study, from alpine wetlands to temperate steppe grasslands, soil nutrients and moisture decreased, and salinization degree increased, which lead to the deterioration of the environmental (Table 1), but the associations between grasses and microorganisms in these grasslands remained tight, as shown by the higher connectivity and robustness. Interactions between plants and soil microbes have been shown to influence plants, where the positive effect of plants and microbes mainly affects the advantage of the plant species, as microbial symbionts and other growth-promoting soil microorganisms intensify interspecific competition (Bever, 2003). This is a good explanation for the decrease in grass diversity from wetland to temperate steppe in our study, which was caused by fewer and fewer dominant species in grassland vegetation from alpine wetland to temperate steppe. Enhanced plant productivity and diversity could strengthen the heterogeneity of the soil environmental, further increasing the connection of the microbial network, which in turn could promote the sustainable development of grassland. In our study, we used partial Mantel tests to demonstrate the correlations between grass productivity and diversity with grass-microbial ecological networks (Table 2). Combining soil biodiversity with plant diversity increased the predictive power of biodiversity on ecosystem stability (Chen et al., 2021). Our study is unique in that we simultaneously considered the influences of both above- and below-ground biodiversity as well as the interaction. In different soil taxa and the interaction of the ground grass, fungal community and ground grass was more stable (Figures 3I, J), and the soil variables for the influence of the above- and below-ground biological interaction is less than the effect of grass productivity and diversity (Table 2). Altogether, our findings suggested that there a stable and complex interaction network is formed between grass and soil microbial community.

## 5. Conclusion

Revealing the relationship between soil microbial communities and grassland grasses in the Qinghai-Tibet Plateau is of great significance for promoting the sustainable development of grassland grasses and grassland management. In this study, samples were collected over a spatial scale of 2,121 km from the Three-River Headwaters region of the Qinghai-Tibet Plateau to study the microbial community changes of four types of grasslands: alpine swamp meadows, alpine meadows, alpine steppe and temperate steppe, as well as the relationship between grass productivity, diversity, soil variables and microbial community. There were positive correlations between grass productivity and archaea and bacteria α diversity, and negative correlations between grass diversity and archaea richness and Shannon, and bacteria and fungi α diversity. Moreover, an increase in microbial β-diversity was observed along with increased discrepancy in grass diversity and productivity and soil variables, but the contribution of grass productivity on microbial community was stronger than that of soil variables and grass diversity, which implies that microbial community was more related to grass productivity. IDENs analysis indicated that the grass species formed complex and stable ecological networks with some bacterial, archaeal, and fungal species, and the connection are all positive. Besides, there was a significant correlation between grass productivity and grass-microbe interactions. These results will help us better understand the interaction between soil microorganisms and grasses in alpine grassland ecosystems, and are of great significance for the establishment of regulatory mechanisms between grasses and microorganisms in alpine grassland ecosystems.

## Data availability statement

The datasets presented in this study can be found in online repositories. The names of the repository/repositories and accession number(s) can be found in the article/Supplementary material.

## Author contributions

GL, JW, and HZ conceived and designed the experiments. ND, SY, and XJ performed the experiments. YW, KF, LW, and SG analyzed the data. YW and YD wrote the paper. All authors read and approved the final manuscript.
