# Supplementary material for: Grass-microbial inter-domain ecological networks associated with alpine grassland productivity
Source: Front Microbiol. 2023 Jan 25;14:1109128. doi: 10.3389/fmicb.2023.1109128 (PMC9905801; doi:10.3389/fmicb.2023.1109128)
Supplement: Supplementary file 1 [file Data_Sheet_1.docx]

Supplemental Information


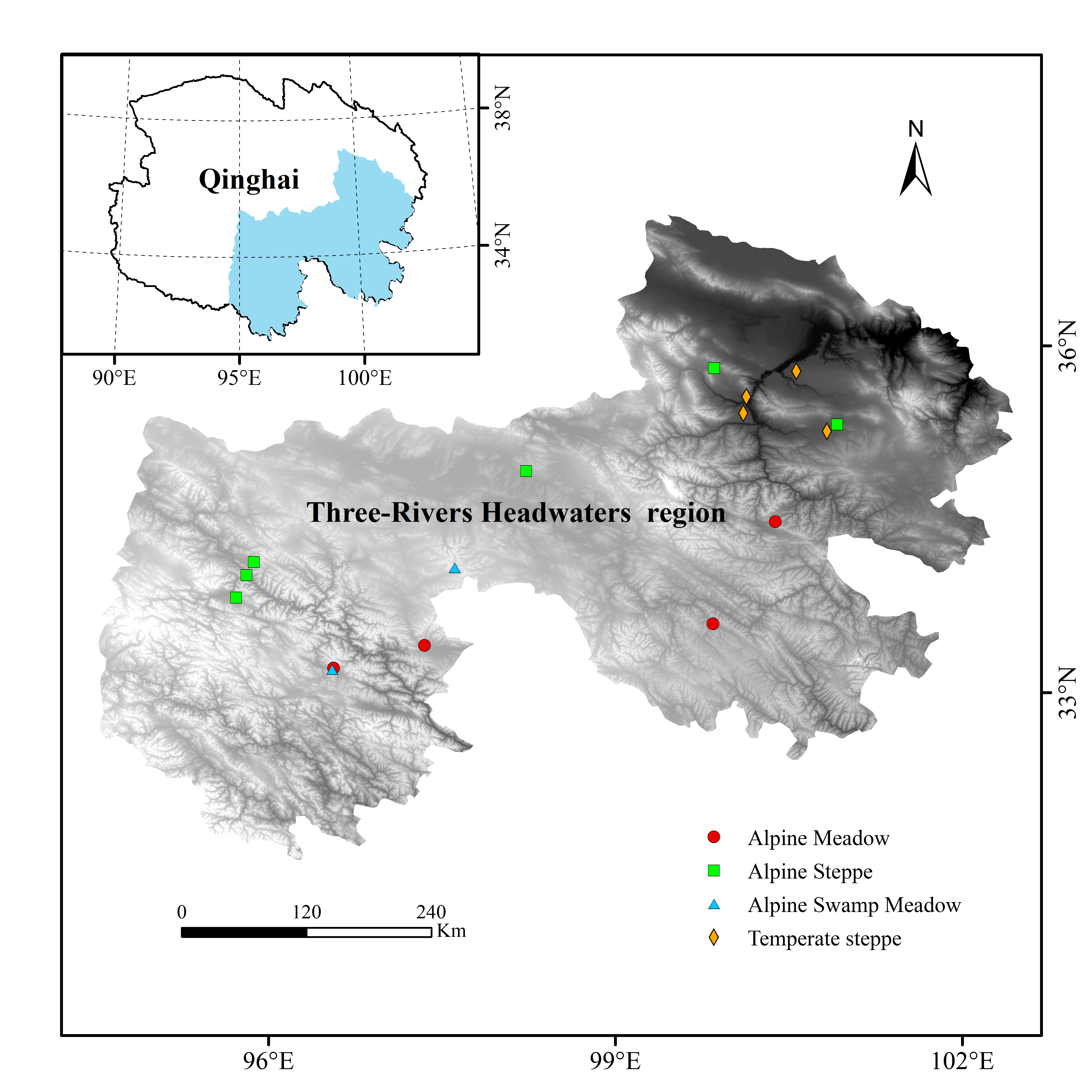
**Fig.S1 Spatial distribution of sampling sites across different grassland types in the Three-Rivers Headwaters region.**


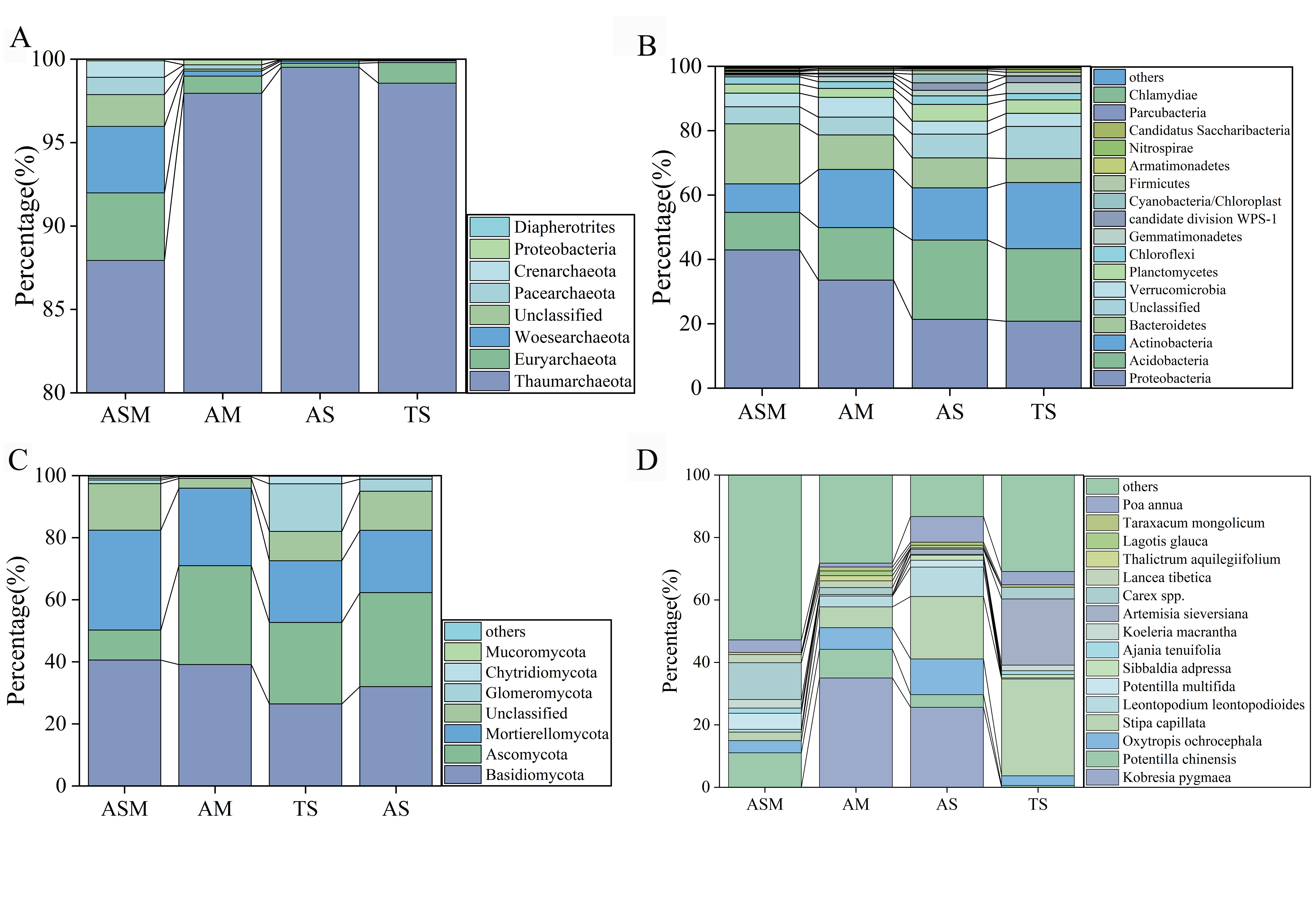
**Fig.S2 Changes in microbial composition in different grassland types.** Phylum level composition of the archaeal (A), bacterial (B), and fungal (C) communities. (D) Species level composition of the grass community.

**Fig.S3 Prediction of microbial functions in different grassland types.** Function based on FAPROTAX for archaea (A) and bacteria (B). (C) Fungal function based on FUNGuild.


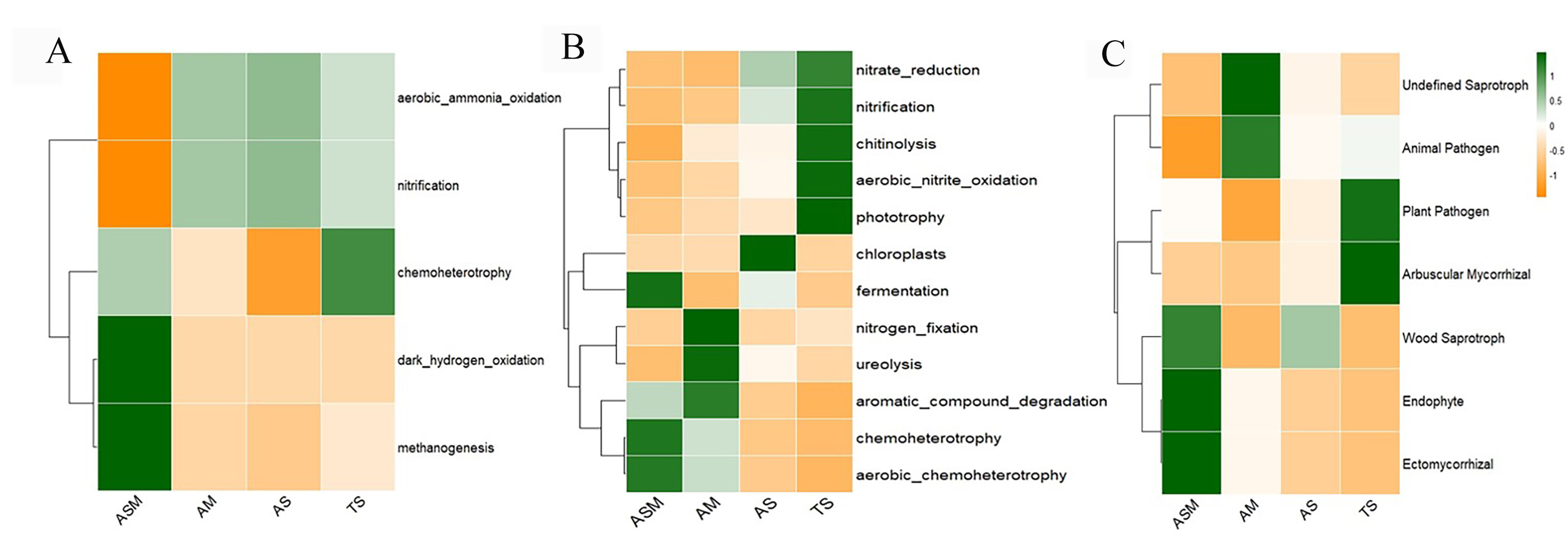


**Fig.S4 Relationship between microbial and grass community structure (NMDS) and environmental factors in different grassland types (CCA).** NMDS of archaea (A), bacteria (B), fungi (C), and grasses (D). CCA of archaea (E), bacteria (F), fungi (G), and grasses (H). The Soil pH **(**pH**)**, total nitrogen (TN), total phosphorus (TP), soil moisture content (SMC), the grass coverage degree (CD), species(S), dry biomass (DB), and the mean annual temperature (MAT). Significant at the 0.05(*) and 0.01 (**) level.


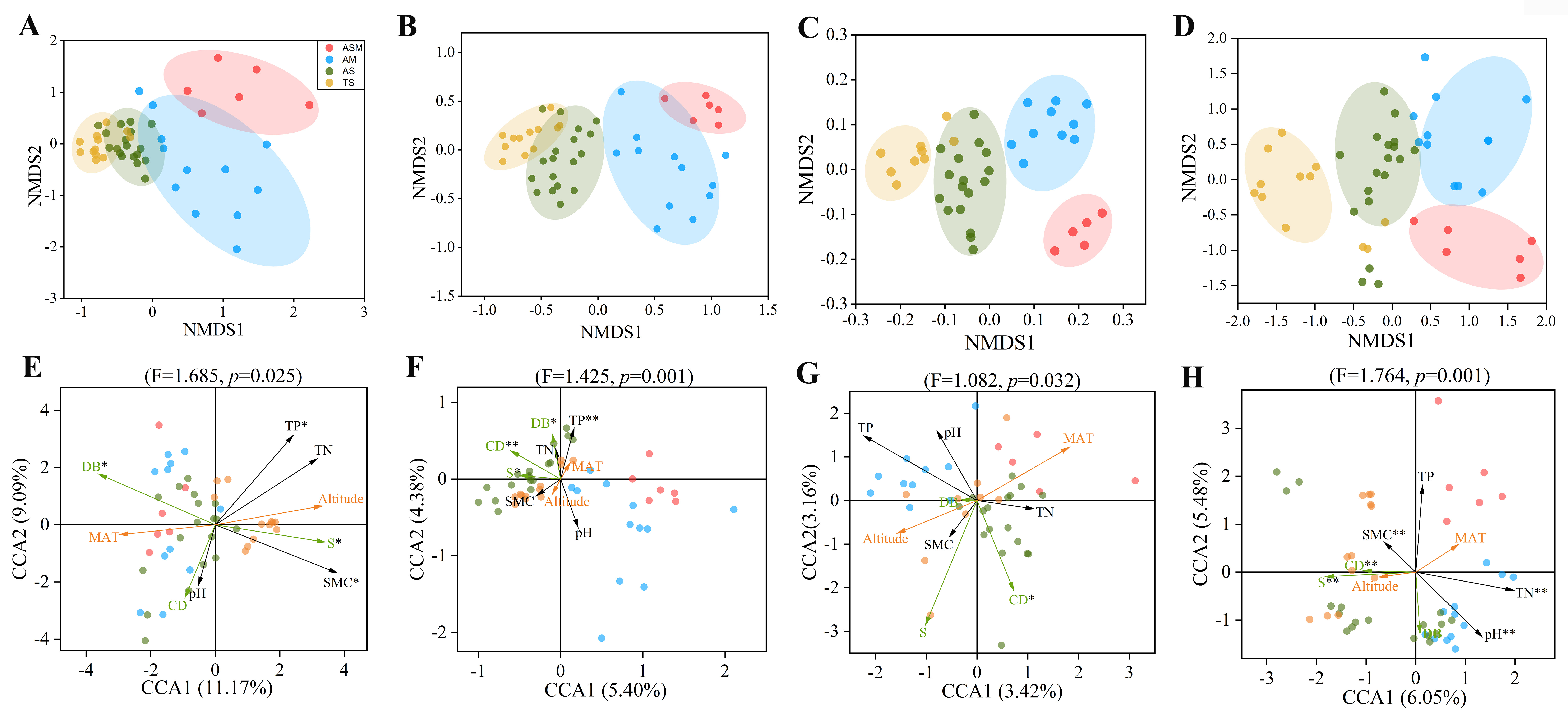


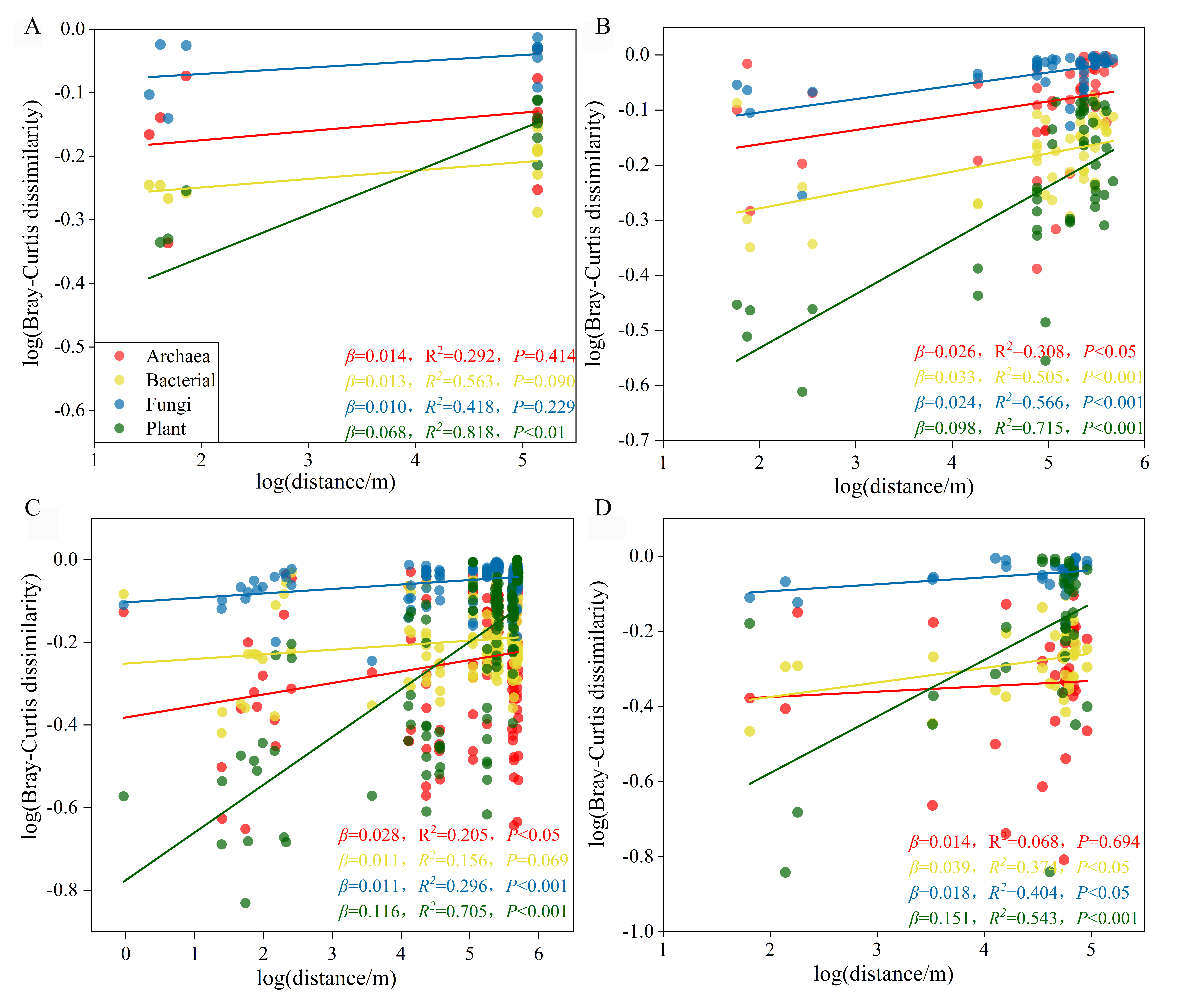
**Fig.S5 Spatial conversion rate of microorganisms in different grassland types.** (A) Alpine wetland meadow (ASM); (B) Alpine meadow (AM); (C) Alpine steppe (AS); (D) Temperate steppe (TS).

**Fig.S6 Network interactions between different microorganisms.** (A) The interactions of bacterial and archaeal. (B) The interactions of bacterial and fungal. (C) The interactions of archaeal and fungal. Robustness of archaeal-bacterial (D), bacterial-fungal (E), and archaeal-fungal (F) networks.


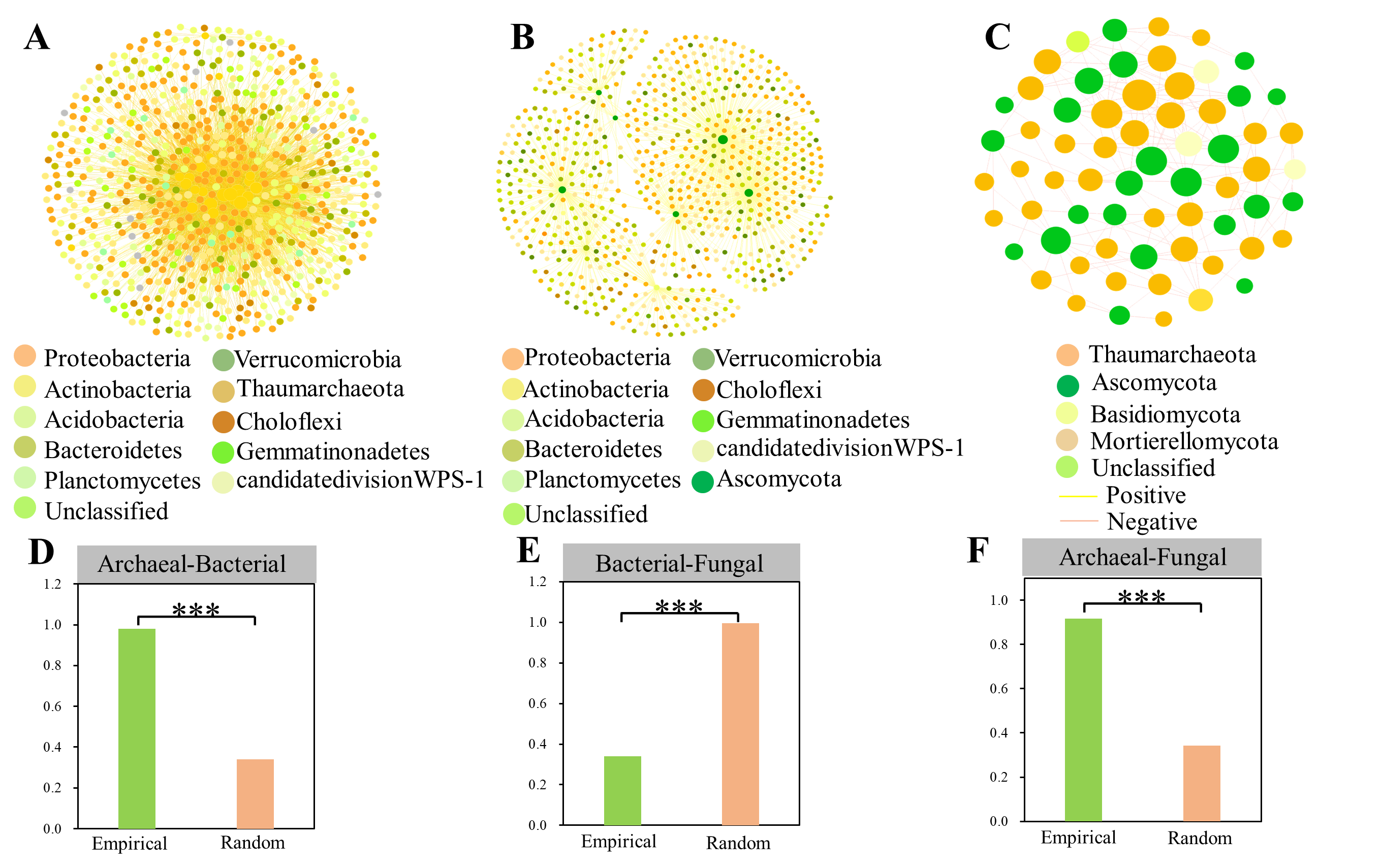


**Table S1 Relationship between soil variables and microbial α diversity, grass α diversity, and grass productivity.**

| Soil variables | | pH | CEC | OM | TN | TP | TC | SMC |
| --- | --- | --- | --- | --- | --- | --- | --- | --- |
| Archaea | Shannon | -0.481*** | 0.515*** | 0.369** | 0.418** | 0.476*** | 0.286* | 0.258 |
|  | Richness | -0.517*** | 0.465*** | 0.506*** | 0.544*** | 0.407** | 0.337* | 0.164 |
|  | Pielou | 0.046 | -0.005 | -0.098 | -0.129 | 0.051 | -0.022 | 0.092 |
| Bacteria | Shannon | -0.586*** | 0.537*** | 0.496*** | 0.495*** | 0.382** | 0.369** | 0.258 |
|  | Richness | -0.222 | 0.157 | 0.224 | 0.243 | 0.254 | 0.038 | 0.006 |
|  | Pielou | -0.363* | 0.347* | 0.232 | 0.186 | 0.167 | 0.309* | 0.224 |
| Fungi | Shannon | 0.105 | -0.001 | -0.085 | -0.067 | 0.001 | -0.192 | 0.009 |
|  | Richness | -0.081 | 0.187 | -0.005 | 0.102 | 0.06 | -0.163 | 0.147 |
|  | Pielou | 0.146 | -0.057 | -0.096 | -0.109 | -0.019 | -0.187 | -0.022 |
| Grass diversity | Margalef | 0.403** | -0.376** | -0.372** | -0.399 | -0.150 | 0.201 | -0.426** |
|  | Shannon | 0.454** | -0.408** | -0.361* | -0.433** | -0.257 | 0.181 | -0.519*** |
|  | Pielou | 0.428** | -0.323* | -0.235 | -0.381** | -0.339* | 0.084 | -0.542*** |
| Grass productivity | Height | 0.329* | -0.270 | -0.369** | -0.256 | 0.156 | -0.007 | -0.159 |
|  | CD | -0.667*** | 0.341* | 0.393** | 0.461*** | 0.050 | -0.240 | 0.756*** |
|  | S | -0.171 | 0.057 | 0.021 | -0.075 | 0.464*** | 0.944*** | 0.028 |
|  | FB | -0.401** | 0.024 | 0.116 | 0.215 | 0.062 | -0.167 | 0.329* |
|  | DB | -0.179 | -0.165 | -0.135 | -0.098 | 0.169 | 0.417** | 0.168 |

Soil variables were significantly correlated with microbial diversity, grass diversity, and productivity at the 0.05*, 0.01**, 0.001***.

**Table S2** Changes of archaea, bacteria and fungi community composition in different grassland types.

| Bacteria | | | Archaea | | | Fungi | | |
| --- | --- | --- | --- | --- | --- | --- | --- | --- |
| Phylum | mean | *p* | Phylum | mean | *p* | Phylum | mean | *p* |
| Proteobacteria | 29.65 | 0.011 | Thaumarchaeota | 95.99 | 0.000 | Basidiomycota | 34.52 | 0.002 |
| Acidobacteria | 18.80 | 0.008 | Euryarchaeota | 1.64 | 0.144 | Ascomycota | 24.51 | 0.017 |
| Actinobacteria | 15.93 | 0.008 | Woesearchaeota | 1.13 | 0.319 | Mortierellomycota | 24.28 | 0.004 |
| Bacteroidetes | 11.54 | 0.018 | Unclassified | 0.53 | 0.329 | Unclassified | 10.04 | 0.030 |
| Unclassified | 7.03 | 0.007 | Pacearchaeota | 0.34 | 0.247 | Glomeromycota | 5.26 | 0.225 |
| Verrucomicrobia | 4.62 | 0.003 | Crenarchaeota | 0.25 | 0.386 | Chytridiomycota | 1.06 | 0.109 |
| Planctomycetes | 3.75 | 0.008 | Proteobacteria | 0.10 | 0.239 | Mucoromycota | 0.22 | 0.182 |
| Chloroflexi | 2.24 | 0.001 | Diapherotrites | 0.01 | 0.292 | others | 0.11 | 0.119 |
| Gemmatimonadetes | 1.82 | 0.054 |  |  |  |  |  |  |
| candidate division WPS-1 | 1.38 | 0.060 |  |  |  |  |  |  |
| Cyanobacteria/Chloroplast | 0.82 | 0.288 |  |  |  |  |  |  |
| Firmicutes | 0.91 | 0.009 |  |  |  |  |  |  |
| Armatimonadetes | 0.48 | 0.021 |  |  |  |  |  |  |
| Nitrospirae | 0.29 | 0.037 |  |  |  |  |  |  |
| Candidatus Saccharibacteria | 0.32 | 0.017 |  |  |  |  |  |  |
| Parcubacteria | 0.10 | 0.012 |  |  |  |  |  |  |
| Chlamydiae | 0.09 | 0.147 |  |  |  |  |  |  |
| others | 0.22 | 0.006 |  |  |  |  |  |  |

**Table S3** Dissimilarity test (PERMANOVA) based on Bray-Curtis of archaea, bacteria, fungi and grass communities in soils from different grassland types.

| Dissimilarity | Archaea | | Bacteria | | Fungi | | Grass | |
| --- | --- | --- | --- | --- | --- | --- | --- | --- |
|  | Bray-Curtis | Jaccard | Bray-Curtis | Jaccard | Bray-Curtis | Jaccard | Bray-Curtis | Jaccard |
| ASM vs AM | 1.644 | 1.609* | 6.107*** | 2.409*** | 0.901*** | 0.885** | 9.222*** | 4.726*** |
| ASM vs AS | 9.853*** | 7.068*** | 7.211*** | 5.128*** | 0.871*** | 0.879*** | 7.538*** | 6.514*** |
| ASM vs TS | 13.560*** | 7.580*** | 11.176*** | 6.651*** | 0.874*** | 0.862*** | 6.791*** | 6.140*** |
| AM vs AS | 7.271*** | 5.537*** | 6.107*** | 4.746*** | 0.887*** | 0.881*** | 3.685** | 3.329** |
| AM vs TS | 10.677*** | 6.336*** | 8.644*** | 6.167*** | 0.896*** | 0.870*** | 9.900*** | 6.128*** |
| AS vs TS | 6.789*** | 3.320*** | 3.381*** | 2.976*** | 0.872** | 0.869*** | 4.792*** | 5.288*** |
| WHOLE | 7.795*** | 5.109*** | 6.329*** | 4.593*** | 0.883*** | 0.875*** | 6.486*** | 5.210*** |

The community structure of the two grassland types was significantly different at 0.001***, 0.01** level.

**Table S4 Bipartite networks attribute feature table.**

|  | Grass-Microbe | Grass-Archaea | Grass-Bacteria | Grass-Fungi | Archaea-Bacteria | Bacteria-Fungi | Archaea-Fungi |
| --- | --- | --- | --- | --- | --- | --- | --- |
| No.grasses | 61 | 46 | 63 | 21 | — | — | — |
| No.archaea | 20 | 31 | — | — | 25 | — | 43 |
| No.bacteria | 686 | — | 685 | — | 718 | 749 | — |
| No.fungi | 1 | — | — | 23 | — | 9 | 15 |
| Total links | 4094 | 437 | 3534 | 55 | 4745 | 1149 | 339 |
| Connectivity | 0.500 | 0.516 | 0.500 | 0.522 | 0.018 | 0.501 | 0.201 |
| Web asymmetry | -0.994 | -0.879 | -0.994 | -0.84 | -0.006 | -0.995 | 0 |
| Links per species | 0.997 | 0.970 | 0.997 | 0.960 | 6.638 | 0.999 | 5.828 |
| No. compartments | 2 | 1 | 2 | 1 | 2 | 1 | 1 |
| Cluster coefficient | 0.500 | 0.516 | 0.500 | 0.522 | 0.008 | 0.501 | 0.19 |
| Nestedness | 35.120 | 25.795 | 34.450 | 23.322 | 0.046 | 33.89 | 27.717 |
| Weighted nestedness | 0 | 1 | 0 | 1 | 0.963 | 1 | 0.137 |
| Specialization asymmetry | 0 | -1 | 0 | -1 | -0.009 | -1 | 0 |
| Modularity | 0.048 | 0.058 | 0.020 | 0.076 | 0.07 | 0.003 | 0.213 |
| No. modules | 85 | 49 | 66 | 24 | 72 | 12 | 46 |

**Data Accessibility**

The row sequence data from this study were deposited in the CNCB database with the study accession number PRJCA012847 that are publicly accessible at https://ngdc.cncb.ac.cn/gsub.
